# Integrating regional conservation priorities for multiple objectives into national policy

**DOI:** 10.1038/ncomms9208

**Published:** 2015-09-14

**Authors:** Maria Beger, Jennifer McGowan, Eric A. Treml, Alison L. Green, Alan T. White, Nicholas H. Wolff, Carissa J. Klein, Peter J. Mumby, Hugh P. Possingham

**Affiliations:** 1 Australian Research Council Centre of Excellence for Environmental Decisions, School of Biological Sciences, The University of Queensland, Brisbane, Queensland 4072, Australia; 2 School of Biosciences, University of Melbourne, Melbourne, Victoria 3010, Australia; 3 Indo-Pacific Division, The Nature Conservancy, 245 Riverside Drive, West End, Queensland 4101, Australia; 4 Indo-Pacific Division, The Nature Conservancy, 923 Nu'uanu Avenue, Honolulu, Hawaii 96817-1539, USA; 5 Marine Spatial Ecology Lab, School of Biological Sciences, The University of Queensland, Brisbane, Queensland 4072, Australia; 6 School of Geography, Planning and Environmental Management, The University of Queensland, Brisbane, Queensland 4072, Australia; 7 Department of Life Sciences, Imperial College London, Silwood Park, Ascot, Berkshire, SL5 7PY England, UK

## Abstract

Multinational conservation initiatives that prioritize investment across a region invariably navigate trade-offs among multiple objectives. It seems logical to focus where several objectives can be achieved efficiently, but such multi-objective hotspots may be ecologically inappropriate, or politically inequitable. Here we devise a framework to facilitate a regionally cohesive set of marine-protected areas driven by national preferences and supported by quantitative conservation prioritization analyses, and illustrate it using the Coral Triangle Initiative. We identify areas important for achieving six objectives to address ecosystem representation, threatened fauna, connectivity and climate change. We expose trade-offs between areas that contribute substantially to several objectives and those meeting one or two objectives extremely well. Hence there are two strategies to guide countries choosing to implement regional goals nationally: multi-objective hotspots and complementary sets of single-objective priorities. This novel framework is applicable to any multilateral or global initiative seeking to apply quantitative information in decision making.

## Abstract (web summary)

Conservation initiatives that span multiple countries often face conflicting national priorities. Here, Beger *et al.* develop a framework for integrating regional priorities and national plans by identifying multi-objective and complementary conservation hotspots, and apply it to the Coral Triangle Initiative.

## Graphical abstract

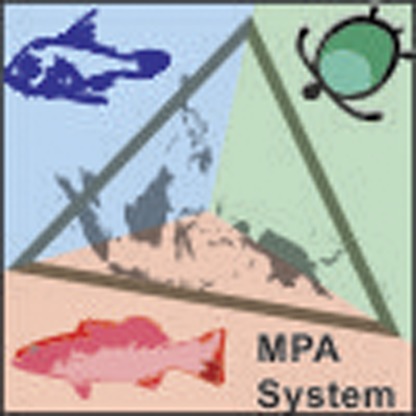

Many regional conservation initiatives try to achieve co-ordinated resource management while recognizing trans-boundary ecological and social processes^1^,^2^,^3^. Evaluating and successfully negotiating the need to meet multiple objectives is pivotal to the success of these often multinational conservation initiatives that need to prioritize investment across a region^4^,^5^,^6^,^7^,^8^. For example, among the more intuitive options is to concentrate efforts in places where multiple conservation benefits can be achieved simultaneously^2^,^9^,^10^, but such multi-objective hotspots may not be ecologically appropriate for some taxa or habitats^11^, or socio-economically equitable^12^. Regionally co-ordinated conservation may achieve broad-scale or even global conservation goals more efficiently^1^,^2^,^3^, but, in reality, almost all resource management actions are established through national, provincial and local planning processes. Missing from most regional efforts are guiding strategies that make trade-offs explicit and offer alternative choices to national planners aiming to consider the broader regional needs.

Examples of multilateral conservation and management efforts are the Micronesia Challenge for marine and terrestrial resource management, Giant Catfish and Irrawaddy Dolphin conservation projects in the Mekong River, Natura2000 in Europe and potential novel land/seascapes in the Arctic and Antarctic. Our case study is the Coral Triangle Initiative on Coral Reefs, Fisheries, and Food Security (CTI-CFF), a regional partnership of six countries: the Philippines, Malaysia, Indonesia, Timor Leste, Papua New Guinea and the Solomon Islands, established to sustain globally imperilled coral reefs and associated ecosystems. This Indo-Pacific region is the global centre of marine diversity, providing ecosystem services such as fisheries and coastal defence for millions of people^13^. Because marine-protected area (MPA) networks are a key management instrument for threatened coral reef ecosystems^14^,^15^,^16^, the CTI-CFF aims to expand their existing Coral Triangle MPA System until at least 20% of it is managed for sustainability and 10% is in no-take protection^13^, an increase from current levels of 14 and 2%, respectively. The broad goals for these investments encapsulate ecological and social considerations including representativeness, connectivity, resilience and the protection of threatened species^13^. These goals aim to secure incomes, livelihoods and food security benefits for coastal communities.

We convert the CTI-CFF goals into six explicit regional conservation objectives (each of which has a short name): represent marine habitats (representation); preserve critical fish spawning aggregation sites for groupers (fish spawning aggregations (FSA)) and important habitats for threatened sea turtles (turtle); maximize larval dispersal among reefs for coral trout (trout) and sea cucumbers (cucumber); and protect reefs likely to experience less mortality from predicted climate change impacts (climate). While accounting for existing no-take protected areas, we identify conservation benefits across the region for each objective using a spatial systematic prioritization tool^17^. Sites with high regional conservation benefits are identified as areas of broad conservation interest that should be regional investment priorities. We therefore report results for provinces that are administrative units on land that have an offshore jurisdiction. We estimate the conservation benefit of each province as the proportion of planning units (10 × 10 km size) that are selected at least half the time in possible solutions to the problem for one or more of the six conservation objectives. We discover eight multi-objective hotspot provinces that achieve conservation benefits in the top quartile for each objective relative to other provinces. However, prioritizing multi-objective provinces will ignore provinces that achieve single objectives effectively and efficiently. An alternative strategy is to target a suite of complementary provinces each with exceptional benefits for one or two objectives. Both strategies need to recognize the importance of every nation reaching its own goals, and contributing to regional goals: to address this, we devise a framework to facilitate a regionally cohesive set of MPAs through regional collaboration, which is driven by national preferences.

## Results and Discussion

### From goals to objectives for the Coral Triangle Initiative

The six objectives considered here arise from regional goals set during the CTI-CFF negotiations and available social and ecological information across the region (Supplementary Table 1). The objectives also encompass ecological MPA network design criteria needed to ensure biodiversity persistence^8^. First, habitat representation is the basic conservation approach to capture all biodiversity^18^ (Table 1). Second, FSA and sea turtles are examples of processes and threatened species that play key roles in food provisioning, tourism and culture, and encompass cross-boundary dependencies. For example, the protection of FSAs is critical for creating a pool of larvae that crosses and recruits across national boundaries^19^. Hence, our design criteria aim to protect all aggregation sites and parts of their land-based catchments^20^ (Table 1; Supplementary Fig. 2a). Sea turtles are emblematic of the challenges faced by threatened migratory species that move long distances between nesting and foraging habitats. Records of long-distance marine turtle migration highlight movements that connect nations and span continents^21^, exposing them to high mortality from artisanal catches and incidental commercial fishing by-catch. We develop a novel planning method to use satellite tracks for four species of marine turtle to maximize connectivity between their nesting and foraging grounds (Table 1; Supplementary Figs 1b and 3c).

Connectivity through larval dispersal underpins recruitment and ecosystem resilience in marine systems where most organisms undergo a pelagic stage^22^. We model demographic connectivity among reefs with a biophysical dispersal model^23^ using specific parameters for coral trout (*Plectropomus leopardus*) and a sea cucumber (*Holothuria whitmaei*). These species were chosen in consultation with representatives from all six countries as important fisheries species with contrasting dispersal abilities (Supplementary Fig. 2c,d). When we attempt to maximize connectivity for individual species, or species groups^24^, the conservation benefits of different locations varies markedly (Fig. 2; Supplementary Fig. 3d,e). Provinces with the highest conservation benefit for larval connectivity objectives are very different to priority provinces for any other objective (Fig. 2a).

Finally, the CTI-CFF goals include mitigating the impacts^4^ of climate change, because the impact of thermal stress on coral reefs is expected to be severe^15^,^25^. Protection cannot directly mitigate climate change stressors, but it serves to enhance recovery processes^6^,^14^,^16^. We identify areas of high conservation benefit by representing reef habitats in 2030 using predicted degradation rates, a novel approach to include explicit climate change objectives into spatial prioritization. We modelled the degradation rates across the coral triangle from dynamic growth trajectories of coral cover over time as a function of predicted thermal stress exposure and aragonite saturation^26^ (Supplementary Fig. 2e). We then degraded the existing coral reef habitat according to predicted rates to serve as conservation targets in this analysis.

### Reconciling conservation benefits for multiple objectives

**Figure 1:**
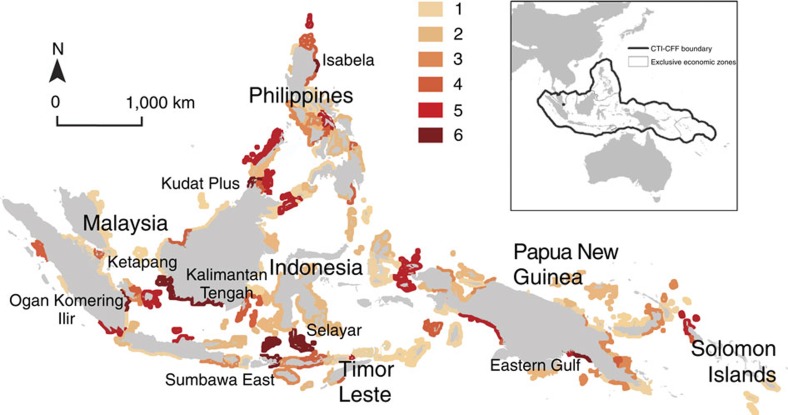
Multi-objective conservation benefit hotspots in the Coral Triangle. Map summarizing the outcomes of objective-driven spatial conservation planning for six distinct conservation objectives in the Coral Triangle. Provinces with high conservation benefit (upper quartile) for every one of the six objectives are shown in dark red with graduated colours showing provinces that achieve conservation benefits for 5 down to 1 objectives. Some of the provinces we highlight as multi-objective hotspots are already areas of interest to the CTI-CFF (for example, Northern Sabah in Malaysia), while others emerged from our analysis (Ketapang and Kalimantan Tengah, and Selayar in Indonesia; Isabela in the Philippines; Eastern Gulf in PNG).

**Figure 2:**
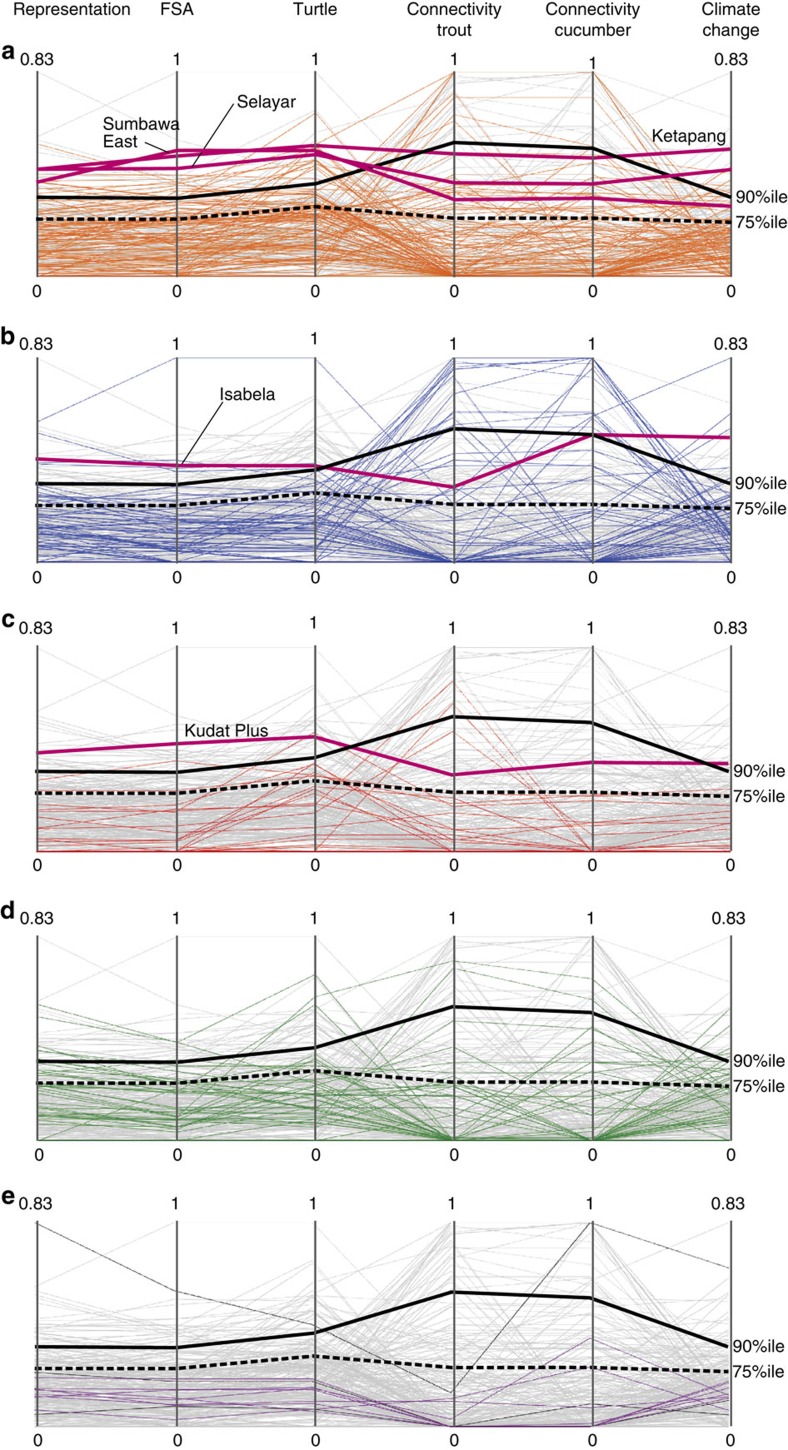
Conservation benefit for each province for each of six objectives. Parallel coordinates plot displaying conservation benefit (ranging from 0 to the maximum) for six conservation objectives on their axes: representation, fish spawning aggregations (FSA), sea turtle habitat and migration (turtle), larval dispersal connectivity for coral trout (connectivity trout) and sea cucumbers (connectivity cucumber), and climate-driven habitat degradation (climate change). Each coloured line represents conservation benefit of provinces for the countries of: (**a**) Indonesia, (**b**) Philippines, (**c**) Malaysia, (**d**) Papua New Guinea and (**e**) Solomon Islands (purple) and East Timor (black), with other Coral Triangle provinces in the background (grey). Black lines denote the conservation benefit threshold for multi-objective hotspots (upper quartile, dashed line) and highest conservation benefit (90 percentile, solid line). Red lines represent examples of multi-objective hotspot provinces.

When we use different conservation objectives the best locations for marine reserves changes a lot (for example, see Supplementary Fig. 3), especially when underlying ecological processes are heterogeneously distributed. The resulting trade-offs may hamper conservation progress at regional scales, where transaction costs to reach agreement among collaborating institutions could outweigh potential benefits. These issues are difficult to overcome for conservation initiatives, as missing from most regional efforts are guiding strategies that make trade-offs explicit and offer alternative choices to national planners aiming to consider regional needs. Ideally, conservation efforts are implemented in places where they can achieve the highest benefits for multiple objectives simultaneously^5^,^12^. In the CTI-CFF, we discover eight provinces that achieve high conservation benefit for all six objectives (above the upper quartile selection frequency) (Fig. 1), and call them ‘multi-objective hotspots'. Akin to biodiversity hotspots, which are places where the highest number of species ranges overlap^27^, multi-objective hotspots are provinces where conservation benefits for multiple objectives coincide (Fig. 1). Multi-objective hotspots are a small subset of centrally located provinces, but investing in them alone would conflict with the nature of fair multilateral collaboration and equity^12^. At the same time, the conservation benefit for each objective achieved by multi-objective hotspots invokes quality trade-offs^10^, as the provinces that are best at meeting a single objective are not often part of the multi-objective hotspot portfolio (Fig. 2). Multi-objective hotspots are likely to always exist in heterogeneous planning problems, as they embody the trade-off between the level of benefit and achieving many objectives at once.

**Figure 3:**
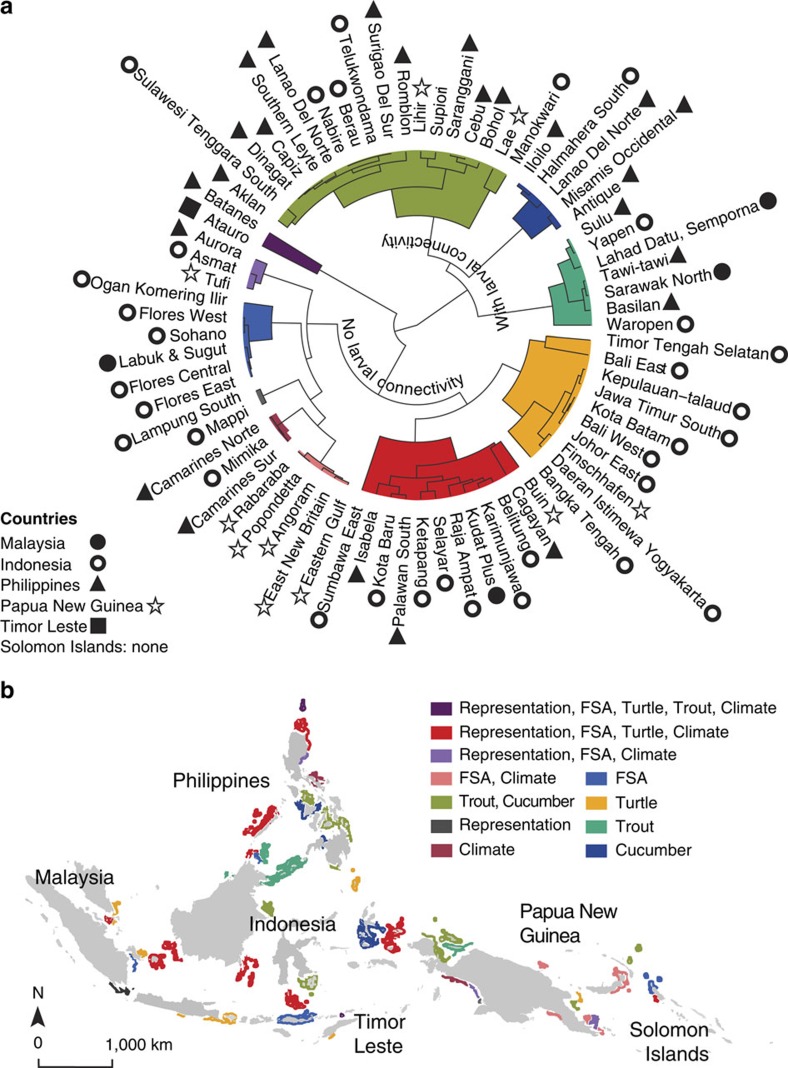
Complementary maximum high conservation benefit provinces for single objectives. Comparison of provinces with very high conservation benefits (that is, top 10%), for specific objectives: (**a**) dendrogram of Euclidian dissimilarities in the distribution of conservation benefits for six objectives. Provinces group distinctly into clusters that are dominated by highest conservation benefits for single and combined objectives with maximum possible benefits for each objective across the region. (**b**) Provincial map showing the spatial distribution of complementary highest conservation benefits for single or subsets of conservation objectives.

When we identify the provinces with the highest conservation benefits for any single objective (that is, within the upper 10%), we discover typically low performance across two or more of the other objectives (Fig. 2). This illustrates the spatial incongruence of high-priority provinces within and across countries (Fig. 3) and raises the option of a second strategy to achieve the highest conservation benefits, given multiple objectives. We offer a complementarity approach to allocating conservation resources to meet all six objectives across the region (Fig. 3), where highest overall conservation benefits can be secured by coordinating investment in provinces that can generate highest benefits for different objectives. For example, the province of Buin (Papua New Guinea) is very effective at meeting four of six objectives (Fig. 3a), but complementary conservation actions to sustain dispersal connectivity are needed elsewhere (Fig. 3). Investing in complementary conservation actions across a region caters towards the unique aspirations, resources and opportunities of each country. Aiming to capitalize on the highest conservation benefits for certain objectives in different parts of the region, complementary approaches recognize the spatially highly variable nature of conservation assets. For example, diverse processes and environmental conditions drive the habitats frequented by sea turtles^21^, the locations of larval dispersal hubs^23^ and reefs that appear more resilient to climate change impacts^15^,^22^,^25^.

### Co-ordinated prioritization to inform national policy

Co-ordinated multilateral conservation can reduce overall cost when targets can be managed in one location for the benefit of all collaborators^1^,^2^,^3^, such as in multi-objective hotspots. Yet, in most settings, these approaches, while cost-effective, violate principles of equity (of both conservation burden and benefit), local resource stewardship, cultural connectedness to natural resources and risk spreading to counter localized catastrophes. Complementarity strategies, on the contrary, ensure that maximal conservation benefit for each objective is achieved, but depending on the objective targeted, they may be less efficient because they are not achieving multiple objectives in a single location.

**Figure 4:**
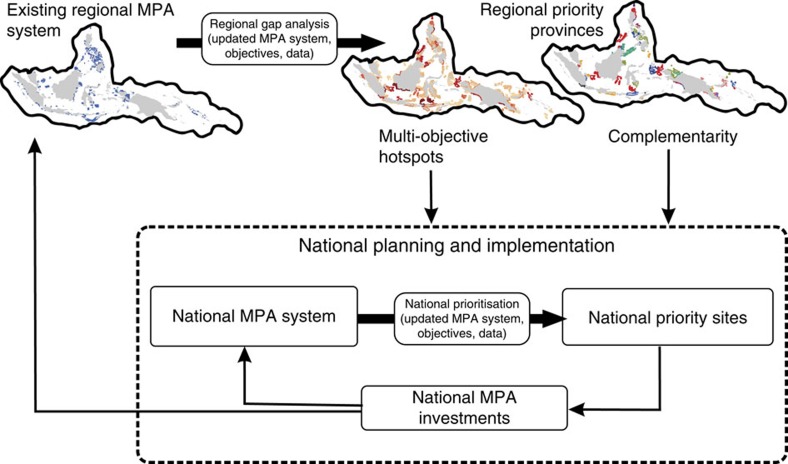
Regional–national conservation framework. Schematic of a dynamic decision-making framework for embedding national priority setting into a regional context, where national goals are chosen with the additional decision criteria of regional priorities in mind. The framework includes scope for adaptive reassessment and planning.

Multilateral initiatives involve sovereign nations whose specific goals and resources govern how the two strategies presented here are implemented. These strategies provide the basis for integrating regional conservation benefits and national planning processes into a dynamic decision framework according to national preferences and resources (Fig. 4). In the Coral Triangle, it can enable the interplay between national goals and governance and regional commitments to the CTI-CFF. At a national scale, countries can utilize the multi-objective hotspot and complementary maps to invest in provinces that meet their national priorities and have regional benefits. Countries with multi-objective hotspots may wish to focus conservation efforts in these provinces, in combination with investing in complementary provinces. Countries lacking multi-objective hotspots can direct national preferences towards complementary provinces that achieve highest regional conservation benefits for targeted objectives. In such a framework, coordination among countries serves to avoid overlooking or under-representing some objectives in the long term, as regional conservation benefits are continually reviewed and updated to capture achievements of independently acting nations meeting their own goals (Fig. 4). Through the coordination of the CTI-CFF, decisions made in each country can be communicated and the process repeated, thus facilitating a regionally coherent MPA system as part of a long-term strategy.

This analysis demonstrates two alternative approaches to dealing with multi-objective trade-offs on a multinational scale in a practical and politically savvy manner. On the basis of objectives identified from regional goals and available data, we also provide a snapshot of broad areas of high potential conservation benefits in the Coral Triangle. Similar trade-offs are likely in other regions where a dynamic decision framework is applicable. This framework applies widely for institutionally nested conservation planning problems, serving to continually adapt broad- and local-scale priority setting processes and to benchmark local contributions towards broad goals.

## Methods

### Conservation prioritization approach

We collated spatial data for biodiversity, socio-economic and climate features from open and closed sources (Supplementary Table 1), and explore regional conservation benefits using the spatial conservation decision support tool Marxan (freely available at http://www.uq.edu.au/marxan/)^17^ using 17, 264 planning units 10 × 10 km in size. Marxan uses a simulated annealing algorithm to implement the objective of achieving user-defined conservation targets such as the amounts of habitat types in protected areas for biodiversity representation and, where applicable, connectivity constraints, while minimizing the overall cost of a protected area system^17^. For example, a conservation goal could be to identify protected area systems that represent 20% of all habitats and leatherback turtle migration pathways with minimal losses to fisheries profit. Management efficiency is modelled by maximizing the spatial compaction and by minimizing the cost of the resulting reserve system. We elected not to pursue spatial compactness as a parameter (excluding connectivity analyses), as it would bias results towards established MPAs (Supplementary Fig. 1a) at the expense of identifying new conservation priorities. In each scenario, 100 runs were performed to assess the spatial variability in conservation priorities in the different solutions found. Hence, we calculate selection frequencies of reef, benthic and mangroves habitats in Marxan as a proxy for conservation priority areas across the 6 countries and 21 ecoregions^28^ of the Coral Triangle.

### Socio-economic cost of conservation

The total annual economic value of coastal and marine habitats in the Coral Triangle (coral reefs, mangroves and sea grass) is an estimated US $2.3 billion for Indonesia and the Philippines alone^29^. Many valued economic activities generating this wealth will be constrained when new protected areas are implemented to protect these ecosystems. Therefore, this analysis considers the human dimension as an indicator of potential conflict with users arising from restricting their activities (for example, prohibiting fishing)^30^. We model them as foregone artisanal fishing profit for coral reef no-take reserves. For mangroves, we use the number of prospective local users (average population density per 10 km^2^ area)^31^ as a proxy for potential conflict arising from mangrove-protected areas. The resulting socio-economic cost index represents the ‘cost' value used in Marxan and covers mangrove and reef habitats (Supplementary Fig. 1b). All else being equal—places with a large cost will be avoided.

### MPA delineation

The countries in the Coral Triangle have successfully established more than 1,900 MPAs (Supplementary Fig. 1a, July 2013)^4^; we needed to define ways of representing these existing MPAs in Marxan. Many locally managed protected areas throughout the region with known boundaries (*n*=498) were often smaller than a single planning unit (10 × 10 km) used in the software. These small areas were assumed to be made up of their designated habitat (reef, sea grass or mangroves) and be entirely no-take. To represent their contribution to the amount of protected habitat they contribute, we calculated the proportion of these protected habitats in planning units, and where the cumulative reserved area per planning unit exceeded 50% of the total planning unit habitat, the planning unit was treated as an existing protected area in our analysis. We further represented no-take areas within large protected areas that encompass multiple planning units (*n*=58, no zoning plans), by randomly selecting 10% of the planning units within to be allocated protected in our Marxan analyses—this number is in the mid-range of no-take amounts typical in the region. This method is fair across all large MPAs, but it possibly over- or underestimates actual habitat protection levels. We used this representation of MPAs in the region to calculate the amounts of protected features currently protected for each country and ecoregion.

### Fish spawning aggregations

The NGO Science and Conservation of Fish Aggregations (SCRFA) has collected FSA data in the region over the past decade (Supplementary Fig. 2a)^20^. The database contained 216 records for 11 aggregating fish families, mostly Serranids (groupers). We aimed to capture 100% of known aggregation sites in reserve systems. To enhance the potential movement of fish from their home territories to the aggregation site, we created a 20-km catchment area and set targets of 50% representation of FSA catchments in conservation areas.

### Connectivity (larvae and turtles)

Marxan's algorithm identified sets of reserve sites fulfilling conservation targets while maximizing connectivity^24^, with the connectivity strength CV_*ij*_ between planning units defined by either larval dispersal or turtle movement. To represent turtle migration connectivity, we developed a novel method to incorporate telemetry data into systematic conservation planning. Connectivity was calculated from the individual tracks of four species of turtle (Supplementary Fig. 2b). Ideally, each part of a track should be protected to ensure a turtle can reach a feeding or breading site. We identified planning units along the turtle's path, and assigned a standardized connectivity strength to each pair of planning units along the track connected by turtle movement. By adding the resulting connectivity matrices for all individual tracks, we obtained accumulated connectivity matrices CV_*ij*_, with higher values for pairs of planning units that were connected by more animals.

Larval dispersal was modelled as connectivity strength among reefs based on a biophysical model of larval dispersal^23^,^32^. Transporting larvae by advection and diffusion processes in surface ocean currents, the dispersal model combined larval biological traits (pelagic larval duration and survival rates) and larval behaviour (for example, homing towards a reef) to obtain the likelihood of larvae moving among reefs^32^. Larval traits for coral trout (*P. leopardus*) and a sea cucumber (*H. whitmaei*) were collated from the literature for pelagic larval duration, pre-competency period, mortality, swimming and homing behaviour, and spawning time (Supplementary Table 2; Fig. 2c,d). We calculated the average connectivity between sites as the proportion of larvae arriving at a site from source reef complexes^23^. Due to the broad regional focus and computational requirements, the biophysical dispersal model is based on reefs that are clustered into contiguous or closely adjacent reef complexes^33^. Subsequently, discrepancies exist in the spatial scale of reef complexes (*n*=425 for entire region) and the scale of reef habitat used in spatial planning. The connectivity among reef complexes was upscaled using the method developed by Beger *et al.*^34^, and the resulting asymmetric connectivity matrices, defined by CV_*ij*_ for flows *l*_*i,j*_ between all pairs of planning units *i* and *j* in Marxan (CV_*ij*_ (ref. 24)), provided directional larval dispersal strengths for *P. leopardus* and *H. whitmaei*.

### Climate change

A coral reef trajectory model was used to evaluate the effects of ocean acidification and rising temperature on living coral, expressed as coral cover^26^. Coral reef trajectories were modelled for each 0.5 × 0.5-degree cell within the study area. Coral cover was initiated at 30% in the year 1865 and estimated annually to 2050. Coral cover dynamics were captured using two ‘typical' Indo-Pacific taxa with branching (*Pocillopora*) and massive (*Porites*) growth forms. Coral cover in each year was predicted from previous years' coral cover plus the combined annual effects of growth and mortality. Mortality was influenced by thermal stress-induced bleaching and growth was influenced by temperature and aragonite effects on calcification. Thermal stress events and aragonite levels were based on global climate model estimates (see below). Because we wanted to model only the impacts of climate change on coral trajectories, we excluded local stressors such as fishing, nutrients and cyclones. Thus, the model essentially integrates the combined spatially variable effects of warming and acidification on the region's corals. The 0.5 × 0.5-degree cell resolution was downscaled to the planning unit resolution of 10 × 10 km by linear interpolation, including coastal areas not modelled in the coral reef trajectory model because they were outside the coarse resolution land mask. Rates of reef decline were calculated as a proportional degradation value from coral cover at present (2010) to that at 2030 (Supplementary Fig. 2e). The rates were used to degrade present coral reef habitats (as used in the rest of the analysis) to obtain new amounts of coral reef habitat per planning unit.

Sea surface temperature (SST) for the years 1865–2055 were based on adjusted output from two AR4 Global Climate Models (CM2.0 and 2.1), using the approach developed by Donner *et al.*^35^ and Donner^36^. AR4 GCMs were used because these models have moderate climate sensitivity and have been used for multiple studies of coral reef futures^36^,^37^,^38^. The projected evolution of SSTs and thermal stress is similar in the recent CMIP5 output^39^. Thermal stress leading to coral bleaching was estimated from the accumulation of degree heating months (DHMs), a metric commonly used in modelling studies to estimate thermal stress responses on coral reefs. The maximum DHM in each year was calculated as maximum 4-month accumulation of SST in excess of the maximum value from the monthly 1985–2000 SST climatology. The probability of DHM >*x* °C·month (where *x*=1, 2, 3, …, 8) was calculated per year using DHM values from a 10-year moving window for all available model runs. Thus, the DHM exceedence probability is calculated from *n*=80 (8 model runs × 10 years) for 1865 through 2000 and *n*=20 (2 model runs × 10 years) for 2001–2050.

The saturation state of aragonite (Ω_ar_) between the years 1860 and 2050 was obtained from the simulations of the University of Victoria Earth System Model (UVic), version 2.8. The same model was used to project future ocean acidification and aragonite saturation state of sea water surrounding coral reefs^40^. Using model simulated fields of dissolved inorganic carbon, alkalinity, SST and salinity, the saturation state of aragonite was computed using the carbonate chemistry routine from the Ocean-Carbon Cycle Model Intercomparison Project (OCMIP), as detailed in Cao and Caldeira^40^. The UVic model has a spatial resolution of 1.8° latitude by 3.6° longitude. The aragonite saturation obtained at this resolution was linearly interpolated to produce annual results at 0.5 × 0.5-degree resolution for use by the coral reef trajectory model.

### Caveats

Our analyses identified additional broad priority areas for MPA expansion with best available data. Some provinces that did not feature as regional conservation priorities may already be sufficiently protected. It is also possible that they were a low priority because of insufficient data, particularly for FSAs, and turtle data, or they are near the edge of the connectivity models. For example, the provinces in the Solomon Islands did not show up as a high priority in this analysis, not because they are not a priority for conservation, but because there are already many protected areas there, there was insufficient data (for example, on FSA) and it was near the edge of the connectivity models. While some processes, such as dispersal and migration, are particularly relevant to multilateral conservation coordination, local action and maintenance of existing protected areas is crucial to fulfil national and indeed, regional and global conservation goals. The regional conservation priorities presented here are a first step towards fully integrating regional, national and local coral reef management actions in a dynamic and adaptive manner to ensure long-term biodiversity and sustainability benefits.

## Additional information

**How to cite this article:** Beger, M. *et al.* Integrating regional conservation priorities for multiple objectives into national policy. *Nat. Commun.* 6:8208 doi: 10.1038/ncomms9208 (2015).

## Supplementary Material

Supplementary Information Supplementary Figures 1-3, Supplementary Tables 1-2 and Supplementary References

## Figures and Tables

**Table 1:** Summary of how the six regional conservation objectives were implemented in the decision support software, showing the proportion of features to be included within the regional MPA system (planning unit targets), whether connectivity is implemented (connections between planning units) and citing papers who developed the approaches used.

**Objective**	**Planning unit targets**	**Connections between planning units**	**Method introduced**
Representation	20% Of 11 habitats	None	17,18
	10% Of 21 bioregions		
Fish spawning aggregations (FSA)	20% Of 11 habitats	None	41, this study
	10% Of 21 bioregions		
	All FSA sites		
	50% FSA 20-km catchments		
Sea turtles	20% Of 11 habitats	Maximize migration route connectivity	This study
	10% Of 21 bioregions		
	50% Nesting habitats		
	50% Foraging habitats		
Coral trout connectivity	20% Of 11 habitats	Maximize larval dispersal connectivity	24
	10% Of 21 bioregions		
Sea cucumber connectivity	20% Of 11 habitats	Maximize larval dispersal connectivity	24
	10% Of 21 bioregions		
Climate change	20% Of 11 habitats in 2030, declined by modelled rates	None	This study
	10% Of 21 bioregions		

MPA, marine-protected area.
