# Supplementary material for: Integrating regional conservation priorities for multiple objectives into national policy
Source: Nat Commun. 2015 Sep 14;6:8208. doi: 10.1038/ncomms9208 (PMC4579602; doi:10.1038/ncomms9208)
Supplement: Supplementary Information — Supplementary Figures 1-3, Supplementary Tables 1-2 and Supplementary References [file ncomms9208-s1.pdf]

Supplementary Figure 1.

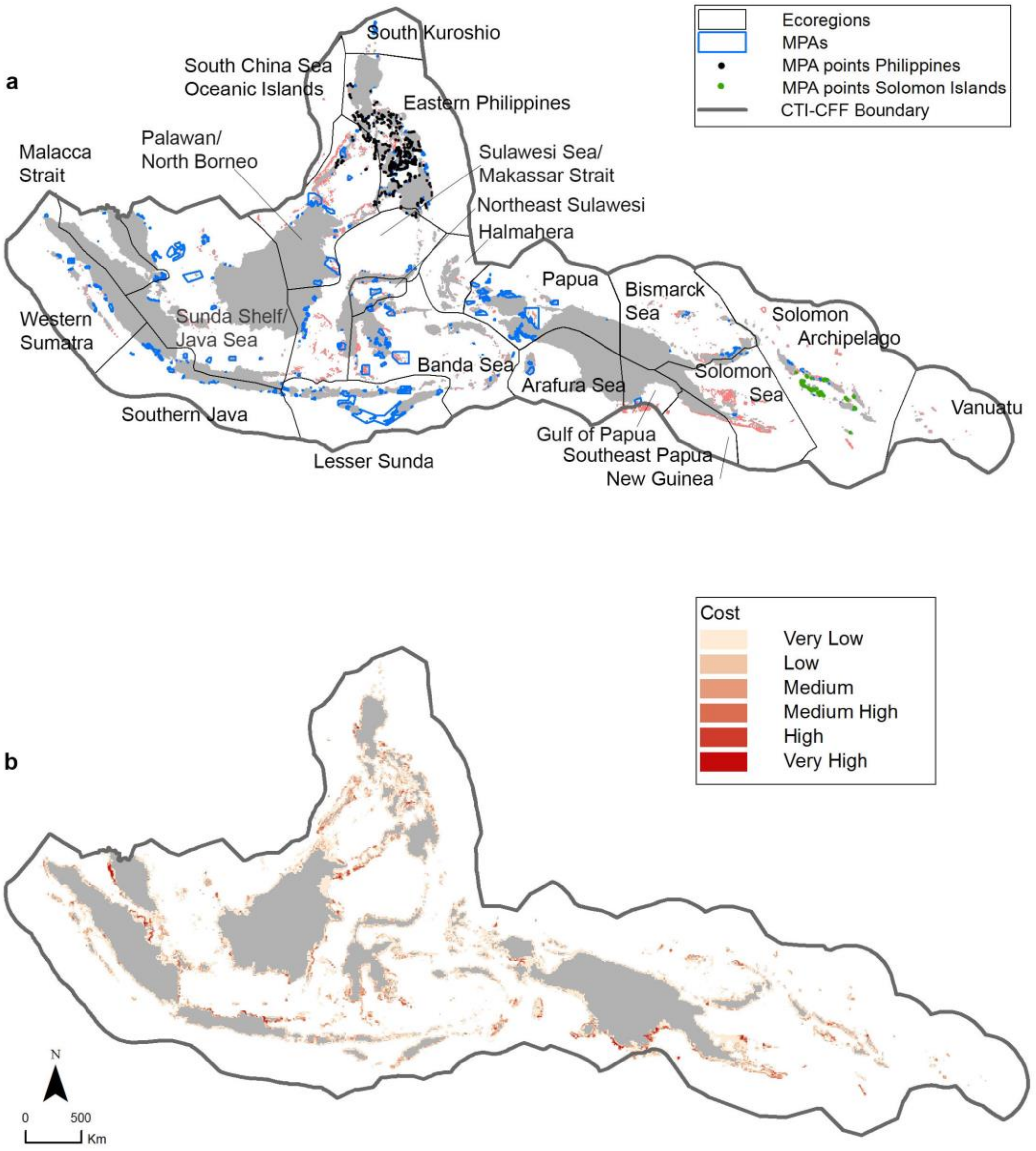

Supplementary Figure 1. Maps of existing MPAs and socio-economic cost. Spatial distribution of a) the CTI-CFF marine protected area system represented in the analysis, showing boundaries of countries and ecoregions, and b) the socio-economic cost index as proxy for foregone fisheries benefit when MPAs are established.

Supplementary Figure 2.

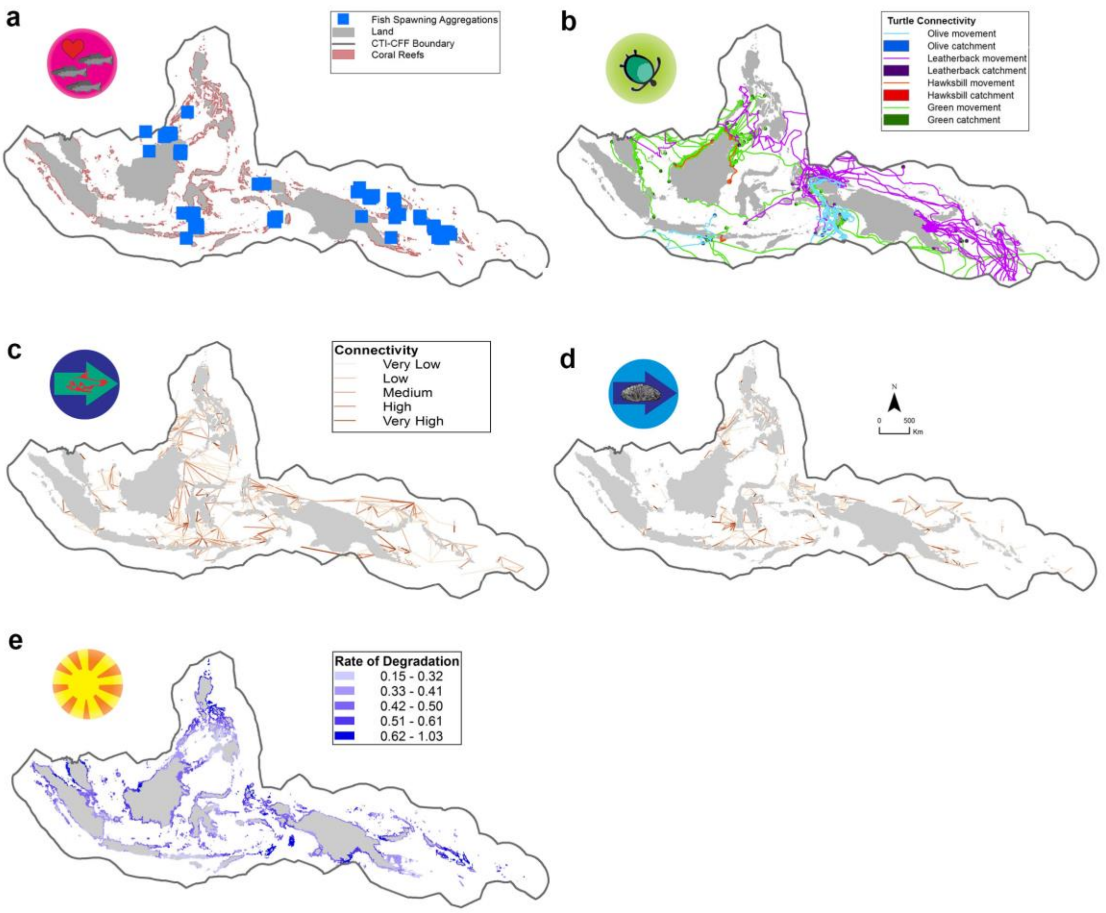

Supplementary Figure 2. Data used for conservation planning. Input data for multi-objective analysis, a) Locations of spawning aggregations (shifted locations for confidentiality), b) Turtle migration tracks of individuals for four sea turtle species, with nesting and foraging sites, c) Proportional larval flow in the coral triangle for Coral Trout *Plectropomus leopardus*, and d) Black Teatfish *Holothuria whitmaei*, capped at above 0.1 (no data for Indian Ocean Sumatra, Malaysian Peninsular, and SW New Guinea), and e) Map of coral decline rates (2010 – 2030) across the Coral Triangle region.

Supplementary Figure 3.

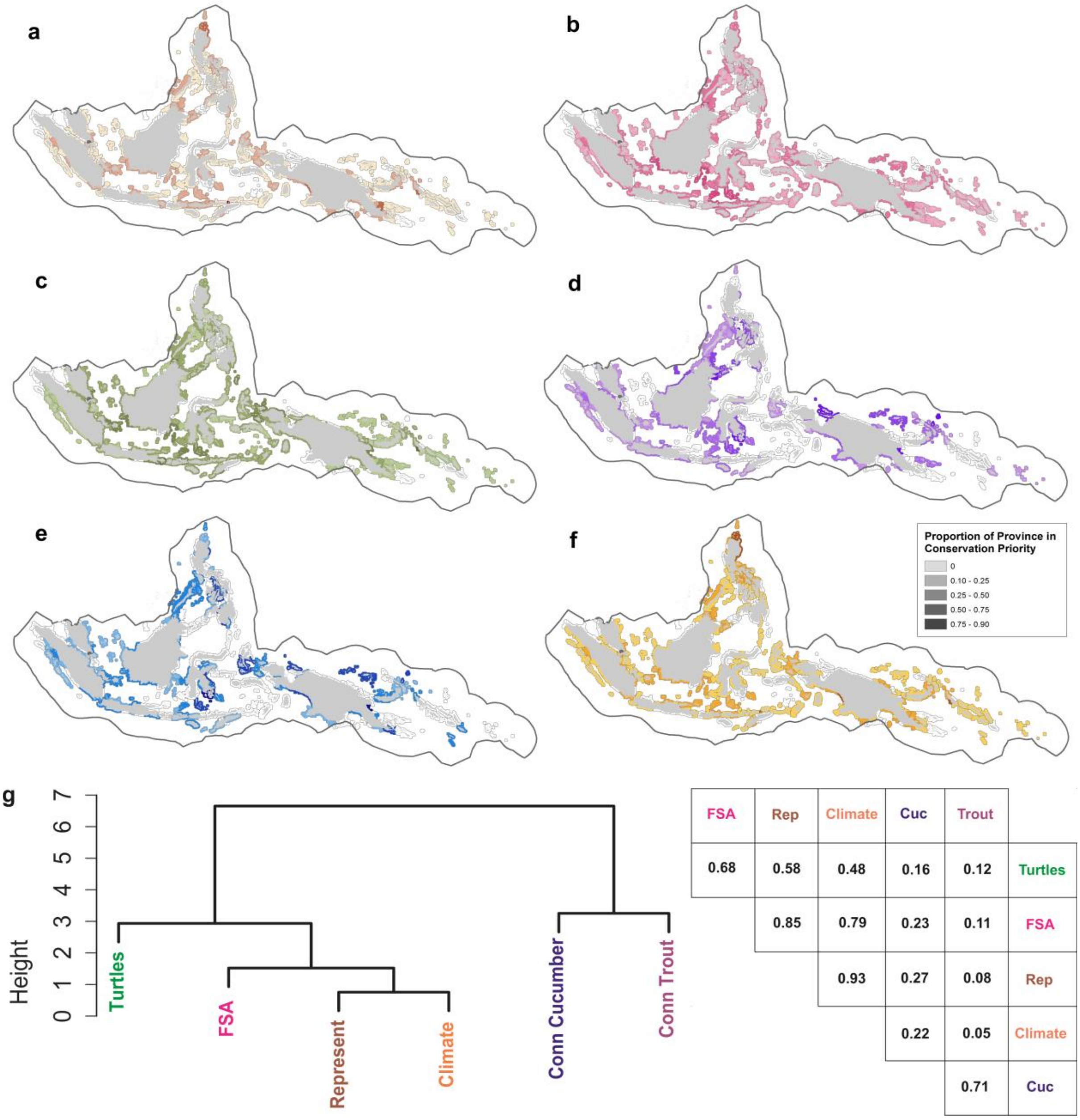

	FSA	Rep	Climate	Cuc	Trout
Turtles	0.68	0.58	0.48	0.16	0.12
FSA		0.85	0.79	0.23	0.11
Rep			0.93	0.27	0.08
Climate				0.22	0.05
Cuc					0.71

Supplementary Figure 3. Maps of conservation priorities. Conservation priorities in the Coral Triangle for multiple objectives of (a) creating a representative reserve system for 11 major habitat types; (b) protecting critical spawning aggregation sites; (c) targeting critical sites and connections for threatened sea turtles; (d) maximizing connections among reefs driven by larval dispersal for groupers (d) and sea cucumbers (e); (f) representing the habitat types in 2030, assuming modelled degradation from climate change, and (g) a dendrogram showing euclidian distances of solutions with inset of correlation values.

Supplementary Table 1. Summary of data sources.

Data layer	Features	Source	Analyses	Reference
Coral reef habitat layers	Coral dense<br>Coral mixed<br>Coral reef slope<br>Rocky reef<br>Seagrass/ algae<br>Sand<br>Mud<br>Other	Download http://coralmap.coremoc.go.jp/	Remove duplicates<br>Remove reefs on land<br>Cut overlapping polygons	[1]
World corals 2010	Corals	WCMC	None	
Mangroves	Mangroves	NASA	None	
Marine bioregions	bioregions	WCMC	None	[2]
MPAs from Coral Triangle Atlas	MPA polygons and points	Download/ agreement	Polygons: readjust some<br>Points: join sources	
MPA points, Solomon Islands	MPA points	Data agreement	Remove duplicates<br>Correct points	Solomon Islands Local Marine Management Areas group
MPA points, Visayas, Philippines	MPA points	Data agreement	Remove duplicates<br>Correct points	[3]
MPA points, Bohol Sea, Philippines	MPA points	Data agreement	none	R Abesamis
MPA points, Philippines	MPA points	Data agreement	Remove duplicates<br>Correct points	University of the Philippines
Artisanal fishing	Opportunity cost for marine areas	NCEAS	none	[4]

World gridded population, 2010	Proxy for opportunity cost in mangrove areas	Download	none	[5]
Turtle sites	5 species	Data agreement	None	WWF-Indonesia, OBIS
Turtle tracks	4 species	Data agreement	Develop connectivity matrices from tracks	WWF-Indonesia, OBIS
Spawning aggregation sites	Aggregation sites for 11 fish families	Data agreement	Site edits to correct location	[6]
Biophysical connectivity	Matrices of mean dispersal strengths among 624 Indo-Pacific sites	Author contribution	Extract connectivity for domain of planning units	[7]
Climate change coral stress	Modelled coral cover for massive *Porites sp.* from present to 2099	Author contribution	Interpolate coral cover for domain, Calculate decline rates	

Supplementary Table 2. Model parameters for the strongly dispersing coral trout and weakly dispersing sea cucumber dispersal connectivity.

	Coral trout (*Plectropomus leopardus*)	Sea cucumber (e.g., commercially important *Holothuria whitmaei*)
Spawning	September to November[8 9]	April and June [10], simulated by two larval release periods per month
Maximum pelagic larval duration	35 days[9]	15 days[11-13]
Competency period	10 days [9]	3 days [10,14]
Homing behaviour	Larvae to settle on nearby reefs if within 10km due to their significant swimming capabilities [15] and auditory and olfactory capabilities [16,17].	Weak swimming capabilities and no homing behaviour [14]

Supplementary References

1 Kakuta, S. *et al.* Satellite-based mapping of coral reefs in East Asia, Micronesia and Melanesia regions. *International Archives of the Photogrammetry Remote Sensing and Spatial Information Science* **XXXVIII**, 534-537 (2010).

2 Spalding, M. D. *et al.* Marine ecoregions of the world: A bioregionalization of coastal and shelf areas. *Bioscience* **57**, 573-583 (2007).

3 Alcala, A. C., Bucol, A. A. & Nillos-Kleiven, P. Directory of marine reserves in the Visayas, Philippines. 178 (Foundation for the Philippine Environment and Silliman University-Angelo King Center for Research and Environmental Management (SUAKCREM), Dumaguete City, Philippines, 2008).

4 Halpern, B. S. *et al.* A global map of human impact on marine ecosystems. *Science* **319**, 948-952 (2008).

5 Center for International Earth Science Information Network (CIESIN)/Columbia University, a. C. I. d. A. T. C. (NASA Socioeconomic Data and Applications Center (SEDAC), Palisades, NY, 2005).

6 Sadovy de Mitcheson, Y. *et al.* A global baseline for spawning aggregations of reef fishes. *Conservation Biology* **22**, 1233-1244, doi:10.1111/j.1523-1739.2008.01020.x (2008).

7 Treml, E. A. *et al.* Reproductive output and duration of the pelagic larval stage determine seascape-wide connectivity of marine populations. *Integrative and Comparative Biology* **52**, 525-537, doi:10.1093/icb/ics101 (2012).

8 Samoilys, M. A. Periodicity of spawning aggregations of coral trout *Plectropomus leopardus* (Pisces: Serranidae) on the northern Great Barrier Reef. *Marine Ecological Progress Series* **160**, 149-159, doi:10.3354/meps160149 (1997).

9 Doherty, P. J., Planes, S. & Mather, P. Gene flow and larval duration in 7 ppecies of fish from the Great-Barrier-Reef. *Ecology* **76**, 2373-2391 (1995).

10 Shiell, G. R. & Uthicke, S. Reproduction of the commercial sea cucumber Holothuria whitmaei Holothuroidea : Aspidochirotida in the Indian and Pacific Ocean regions of Australia. *Marine Biology* **148**, 973-986 (2006).

11 Benzie, J. A. H. & Uthicke, S. Stock size of bêche-de-mer, recruitment patterns and gene flow in black teatfish, and recovery of over-fished black teatfish stocks, on the Great Barrier Reef., (FRDC Project 97/344. Australian Institute of Marine Science, Townsville, 86p., 2003).

12 Uthicke, S. Interactions between sediment-feeders and microalgae on coral reefs: grazing losses versus production enhancement. *Mar. Ecol. Prog. Ser.* **210**, 125-138 (2001).

13 Uthicke, S. & Benzie, J. A. H. A genetic fingerprint recapture technique for measuring growth in 'unmarkable' invertebrates: negative growth in commercially fished holothurians (*Holothuria nobilis*). *Mar. Ecol. Prog. Ser.* **241**, 221-226 (2002).

14 Asha, P. S. & Muthiah, P. Spawning and larval rearing of sea cucumber *Holothuria (Theelothuria) spinifera* Theel. *Bech-de-Mer Information Bullettin* **16**, 11-14 (2002).

15 Leis, J. M. & Carson-Ewart, B. M. In situ swimming speeds of the late pelagic larvae of some Indo-Pacific coral-reef fishes. *Mar. Ecol. Prog. Ser.* **159**, 165-174 (1997).

16 Leis, J. M. Behaviour as input for modelling dispersal of fish larvae: behaviour, biogeography, hydrodynamics, ontogeny, physiology and phylogeny meet hydrography. *Marine Ecology-Progress Series* **347**, 185-193, doi:10.3354/meps06977 (2007).

17 Wright, K. J., Higgs, D. M., Belanger, A. J. & Leis, J. M. Auditory and olfactory abilities of larvae of the Indo-Pacific coral trout *Plectropomus leopardus* (Lacepede) at settlement. *J. Fish Biol.* **72**, 2543-2556, doi:10.1111/j.1095-8649.2008.01864.x (2008).
